# Integrated care for people experiencing homelessness: changes in emergency department use and behavioral health symptom severity

**DOI:** 10.1186/s12913-025-12860-0

**Published:** 2025-05-30

**Authors:** Lexie R. Grove, Justin K. Benzer, Maria F. McNeil, Tim Mercer

**Affiliations:** 1 Department of Population Health, Dell Medical School, University of Texas at Austin, Austin, TX USA; 2 Department of Management, Policy, and Community Health, UTHealth Houston School of Public Health, Austin, TX USA; 3 Implementation Science Institute, UTHealth Houston, Austin, TX USA; 4 Quality Department, Baylor Scott and White Health, Round Rock, TX USA; 5 Department of Internal Medicine, Dell Medical School, University of Texas at Austin, Austin, TX USA; 6 CommUnity Care Federally Qualified Health Centers, Austin, TX USA

**Keywords:** Integrated care, Homelessness, Emergency department use, Behavioral health

## Abstract

**Background:**

Health care for individuals experiencing homelessness is typically fragmented, passive, reactionary, and lacks patient-centeredness. These challenges are exacerbated for people who experience chronic medical conditions in addition to behavioral health conditions. The objective was to evaluate an innovative healthcare delivery model (The Mobile, Medical, and Mental Health Care [M3] Team) for individuals experiencing homelessness who have trimorbid chronic medical conditions, serious mental illness, and substance use disorders.

**Methods:**

We assessed changes in study measures before and after M3 Team enrollment using multi-level mixed-effects generalized linear models. Data sources included primary data collected as part of the program evaluation and administrative records from a regional health information exchange. Program participants continuously enrolled in the M3 Team between August 13, 2019 and February 28, 2022 were included in the evaluation (*N* = 54). The M3 Team integrates primary care, behavioral health care, and services to address health-related social needs (e.g., Supplemental Nutrition Assistance Program benefits and Social Security/Disability benefits). Outcome measures included number and probability of emergency department (ED) visits and behavioral health symptom severity measured using the Behavior and Symptom Identification Scale (BASIS-24) and the Addiction Severity Index (ASI).

**Results:**

M3 Team participants experienced a decrease of 2.332 visits (SE = 1.051, *p* < 0.05) in the predicted number of ED visits in a 12-month follow-up period, as compared to the 12-month pre-enrollment period. M3 Team participants also experienced significant reductions in multiple domains of mental health symptoms and functioning and alcohol and drug use severity.

**Conclusions:**

Individuals experiencing homelessness who received integrated, patient-centered care from the M3 Team saw reductions in ED use and improvements in aspects of self-reported psychosocial functioning and substance use symptoms after enrollment in this novel healthcare delivery model.

## Background

People experiencing homelessness in the United States (U.S.) and other high-income countries face an elevated risk of premature mortality compared to housed individuals and have a substantial burden of psychiatric conditions, substance use disorders, and chronic medical conditions [1]. Available evidence indicates that the prevalence of co-occurring mental health, substance use, and physical health conditions—or trimorbidity—among adults experiencing homelessness is significant and has increased over time; one study using data from a survey of adults experiencing homelessness found that 16.3% of respondents experienced trimorbidity in 2018 compared to 7.9% of respondents in 2000 [2]. Trimorbidity is a risk factor for mortality among unhoused individuals [3].

Despite the high prevalence of complex, often overlapping chronic conditions among people experiencing homelessness, care for this population tends to be reactive rather than proactive, as evidenced by high rates of acute care use. Unhoused individuals have greater rates of emergency department (ED) use [4, 5], and ED users who are unhoused are approximately eight times more likely than those who are housed to have an ED readmission within 30 days [6]. Utilization of public hospitals for mental health conditions and hospital readmission rates are also higher among people experiencing homelessness [7]. Likely driven by more frequent ED use and hospitalizations, unhoused individuals who are covered by Medicaid have been found to have healthcare expenditures that are nearly four times higher than average Medicaid beneficiaries [8].

The status quo of reliance upon acute care to serve people experiencing homelessness inadequately addresses the multiple health needs of this population. In one survey study, nearly 75% of unhoused adult respondents had at least one unmet health need [9]. Unhoused individuals who use the ED and other stakeholders involved in serving these individuals report that ED-based care presents a number of challenges to delivering appropriate care, including lack of care continuity and fragmentation from community resources [10]. However, access to outpatient physical and mental health care among individuals experiencing homelessness is compromised by a number of barriers, such as lack of insurance coverage and transportation [11–13]. When asked in qualitative studies, individuals experiencing homelessness have said that emphasizing continuity of care, including consistent providers and staff, and integrating and coordinating primary care, mental health, and social services together is important [14, 15].

In addition, people who are unhoused may face stigma and discrimination in healthcare settings, which can prevent receipt of needed services and is associated with greater behavioral health symptom severity among unhoused individuals with mental illness [11, 13, 16]. Harm from discriminatory experiences in healthcare settings may be especially acute for people experiencing homelessness who belong to minoritized racial/ethnic groups. Black Americans in particular are starkly overrepresented among people experiencing homelessness, reflecting entrenched patterns of structural racism in the U.S. [17–19]. Unhoused people of color are subject to racial discrimination as well as discrimination based on housing status and/or mental illness [20].

Health services delivery models tailored for individuals experiencing homelessness can address the distinct health challenges faced by this population. To date, such models have largely focused on addressing behavioral health-related needs. In particular, standard case management, assertive community treatment, and critical time intervention have been shown to improve behavioral health symptoms among unhoused individuals and may also reduce hospital use [21]. With respect to physical health, evidence indicates that primary care models designed for people experiencing homelessness, including federally supported Health Care for the Homeless programs, can improve clients’ experience of care and are associated with better quality of chronic condition management, increased primary care use, and decreased ED use [22–25]. However, evidence of delivery models that integrate tailored primary care with evidence-based behavioral healthcare interventions for people experiencing homelessness are lacking.

In this paper, we describe one-year outcomes from a pragmatic, implementation-focused demonstration of a novel integrated care model for individuals experiencing homelessness who have trimorbid conditions—The Mobile, Medical, and Mental Health Care (M3) Team. The M3 Team model provides intensive behavioral health care (for both serious mental illness and substance use disorders), primary care, and services to address health-related social needs to eligible adult clients in Travis County, Texas, integrating services across public mental health and medical health care systems. The program also includes a specific focus on racial equity and prioritizes the recruitment and retention of Black clients. We assess participants’ behavioral health symptom severity and acute care utilization before and after M3 Team enrollment.

## Methods

### M3 team

The Mobile, Medical, and Mental Health Care Team (“M3 Team”) is an integrated community-based team that provides intensive multidisciplinary care to adults experiencing chronic homelessness (i.e., 12 continuous months, or 12 total months over three years with at least four episodes of homelessness) with trimorbidity (chronic medical condition, serious mental illness, and a substance use disorder). Medical care is provided by a physician and nurse employed by a federally qualified health center (FQHC) that is a federal Healthcare for the Homeless (HCH) program grantee providing primary care. Mental health and substance use care, and housing support are provided by staff from Travis County’s Local Mental Health Authority (LMHA), including qualified mental health professional (QMHP) case managers, licensed mental health providers, substance use specialist, a part-time psychiatric nurse practitioner, and a peer support specialist. An academic medical-school-based team provides overall program leadership, support integrating across organizations, and evaluating the care delivery model. The M3 Team goes beyond traditional Health Care for the Homeless (HCH) programs by including comprehensive, evidence-based care for serious mental illness and substance use disorders, integrated with physical health care and social support. The Center for Integrated Healthcare Solutions framework is a typology for classifying care integration along a spectrum ranging from “minimal collaboration” (level one) to “full collaboration in a transformed/merged integrated practice” (level six); using this framework, the M3 Team can be characterized as a level five integrated team with “close collaboration approaching an integrated practice” [26].

The team is mobile in that members bring care to patients (e.g., on the street) in addition to traditional clinic-based care. Enrollment is conducted through referrals, mostly from the partner LMHA and FQHC, but also from community-based street outreach teams. Most (88%) of M3 clients were referred by (i.e., previously enrolled in) the LMHA or FQHC. The team uses evidence-based practices of integrated dual disorder treatment (IDDT), trauma-informed care, housing first, and evidence-based primary care guidelines, contextualized to patients’ living situations and life circumstances. M3 Team service delivery is guided by a patient-centered philosophy based on client choice, and M3 Team providers use a stage of change approach to meet people where they are and in their readiness to address various domains of their health.

### Study design and sample

A retrospective analysis of all program participants continuously enrolled for at least six months in the M3 Team any time between August 13, 2019 and February 28, 2022 (two years, six months; *N* = 54) was conducted as part of an evaluation of the M3 Team. All M3 Team participants were adults living in Travis County, TX who met the following criteria: (1) had at least one chronic medical condition, (2) with co-occurring serious mental illness and a substance use disorder, (3) were experiencing chronic homelessness, and (4) were willing to engage in our integrated care model and consent to treatment. Program participants who had not been enrolled more than six months prior to February 28, 2022, or were discharged prior to completing six months were not included in the analysis. During the three-year study period seven clients graduated, seven died, and seven were lost to follow-up.

### Data sources and variables

This study used primary data collected as part of the program evaluation (i.e., demographics, patient-reported outcomes, and clinician-reported social determinants of health) and administrative records from a regional health information exchange (i.e., emergency department utilization data) [27]. Primary data were collected at least every six months, starting at M3 enrollment (baseline). Data were collected by M3 service providers during dedicated appointments and no incentives were provided. This program evaluation was ruled not human subjects research by the University of Texas at Austin Institutional Review Board.

#### Emergency department use

ED use was measured at 12 months and six months pre- and post-enrollment. Repeated ED visits that occurred within one day of each other were considered as one episode and the earliest visit information was used for the analysis. Program participants who were enrolled at least six months but less than a year were not included in the 12-month measures.

#### Psychiatric symptoms and functioning

Psychiatric symptoms and functioning were self-reported with the Behavior and Symptom Identification Scale (BASIS-24) subscales of relationships, depression and functioning, self-harm, emotional liability, and psychosis. The BASIS-24 is a validated scale that has previously been used to measure psychosocial functioning among individuals experiencing homelessness [28]. Lower scores indicate less difficulty or frequency with the symptom. Reference values (i.e., low, moderate, high) for each subscale were obtained by multiplying the item weights from the BASIS manual by one, two, and three respectively corresponding to responses of a little, moderate, and quite a bit of difficulty.

#### Substance use

Substance use was self-reported with the Addiction Severity Index (ASI) subscales of alcohol use and drug use. Scores range from zero to one, with higher scores indicating greater severity. The ASI manual does not provide reference values (i.e., low, moderate, high). However, a randomized controlled trial for a primary care-based problem drug use intervention provided ASI mean scores by low, intermediate, or severe Drug Abuse Screening Test (DAST-10) scores based on the DAST-10 manual (shown in Table 1 of Roy-Byrne et al. under “Substance use”) [29].

**Table 1:** Participant characteristics, *N* = 54

Variable	*N* (%)
Age (Mean ± SD)	53.13 ± 9.15
Gender	
Male	36 (67)
Female	15 (28)
Other/Missing	3 (6)
Race*	
Black/African American	21 (39)
White	27 (50)
Other/More than one race	6 (11)
Ethnicity	
Hispanic/Latinx	5 (9)
Non-Hispanic/Latinx	49 (91)
Elixhauser Comorbidity Index (Mean ± SD)	2.07 ± 1.78

*The “White” and “Other/More than one race” categories are shown separately here to provide descriptive information about the study sample but were combined into one category in statistical analyses

#### Covariates

Our analyses controlled for demographic variables including a continuous measure of age and categorical measures of race, ethnicity, and gender. In our statistical models, race and ethnicity were dichotomized (Black vs. non-Black, and Latinx vs. non-Latinx) to have large enough sample sizes for analysis, and to represent the M3 emphasis on reducing health disparities for Black program participants.

We controlled for a continuous measure of a medical comorbidity index score, calculated for all clients using the Elixhauser Comorbidity index for outpatient medical care assessed up to one year pre-enrollment and during the enrollment with M3 Team. Our statistical models also included an indicator of whether an encounter or assessment occurred before (COVID = 0) or after (COVID = 1) April 1, 2020, the day after the COVID-19 stay-at-home order was issued by the Texas Governor. Inclusion of this covariate allows us to account for possible changes in ED utilization and behavioral health symptom severity driven by the COVID-19 public health emergency.

### Statistical analysis

To compare ED utilization before and after M3 Team enrollment, we estimated multi-level mixed-effects generalized linear models using the meglm package in Stata 17. We fit these models using random effects at the individual level. We used an unstructured residual structure, which distinctly estimates variances and covariances from the data, to allow for maximum flexibility. We assessed two outcomes: a binary measure of any ED visits and a count of ED visits. We used a logit link and binomial family for the binary outcome and a log link and negative binomial family for the count outcome. We examined changes in outcomes six months pre- and post-enrollment and 12 months pre- and post-enrollment, restricting the sample for each model to individuals with complete information for the relevant baseline and follow-up period. Independent variables were an indicator for time period and control variables described above. We report average marginal effects with delta-method standard errors.

To estimate changes in participants’ mean BASIS-24 and ASI scores, we analyzed repeated measures mixed-effects models using the xtmixed package in Stata 17. We fit these models using random effects at the individual level. We selected an autoregressive residual structure to account for clustering of observations from three time points within individuals (in contrast to ED visit models, which included only two observations per individual). Our primary independent variables were three indicators for time period (six, 12, and 18 months post-M3 Team enrollment, referent = 0 months), and we controlled for demographic characteristics, Elixhauser Comorbidity Index score, and whether the symptom score measurement was taken pre- or post-the start of the COVID-19 pandemic. Control variables account for changes in group composition over time as measurements at each follow-up time point were not available for all participants.

## Results

M3 Team participants had a mean age of 53.13 years (SD = 9.15) and were predominantly male (67%) (Table 1). Black participants comprised 39% of the sample, and 91% were non-Hispanic (91%). Table 2 participants’ mean BASIS-24 and ASI scores were measured upon M3 Team enrollment. Mean scores can be interpreted relative to the reference values provided in Table 2. Mean scores indicate generally moderate behavioral health symptom severity.

**Table 2:** Baseline 24-Item behavior and symptom identification scale and addiction severity index scores, *N* = 54

		Reference values		
Symptom Scale	Mean (SD)	Low severity	Moderate severity	High severity
BASIS-24*				
Depression & Functioning	1.08 (0.37)	0.46	0.91	1.37
Interpersonal Relationships	0.27 (0.13)	0.13	0.27	0.40
Self-Harm	0.09 (0.14)	0.10	0.20	0.30
Emotional Lability	0.39 (0.14)	0.16	0.33	0.49
Psychosis	0.15 (0.10)	0.09	0.19	0.28
ASI^†^				
Alcohol use	0.16 (0.28)	0.08	0.13	0.25
Drug use	0.11 (0.13)	0.06	0.10	0.19

*BASIS-24* 24-Item Behavior and Symptom Identification Scale, *ASI* Addiction Severity Index

*Lower BASIS-24 scores indicate less difficulty or frequency with the symptom, and reference values for each subscale were obtained by multiplying the item weights by one, two, and three respectively corresponding to responses of a little, moderate, and quite a bit of difficulty

^†^Lower ASI Scores, ranging from zero to one, indicate lower severity, and reference values for each subscale were based on baseline ASI scores associated with a low, intermediate, or severe Drug Abuse Screening Test (DAST-10) in a RCT for a primary care-based problem drug use intervention [29]

### Emergency department use

Nearly three-fourths (71%) of participants with complete utilization information had ED use in the six months prior to M3 Team enrollment, and 52% had ED utilization in the six months post-enrollment (Table 3). The mean number of ED visits per participant decreased from 3.15 (SD = 5.11) in the six-month pre-enrollment period to 1.46 (SD = 2.32) in the post-enrollment period (*p* < 0.05). The mean number of ED visits decreased from 4.86 (SD = 7.28) in the 12-month pre-enrollment period to 2.65 (SD = 3.52) in the 12-month post-enrollment period (*p* < 0.05), among participants with complete utilization information.

**Table 3:** Unadjusted emergency department utilization six- and 12-months pre- and post-M3 team enrollment

	6 months^†^	12 months^‡^
Variable	N (%)	N (%)
Pre-M3 Team enrollment		
Had ED visit	34 (71)	32 (74)
Number of ED visits (Mean ± SD)	3.15 ± 5.11	4.86 ± 7.28
Post-M3 Team enrollment		
Had ED visit	25 (52)	28* (65)
Number of ED visits (Mean ± SD)	1.46 ± 2.32*	2.65 ± 3.52*

*ED* emergency department

*p<0.05 in Chi-squared test comparing proportions or paired t-testcomparing means pre- and post-enrollment

^†^*N*=48

^‡^N=43

In adjusted analyses of ED use controlling for model covariates, participants’ predicted probability of having an ED visit decreased by 18.7% points (SE = 8.6, *p* < 0.05) from the six months before enrollment to the six months following enrollment (Table 4). The number of predicted ED visits decreased by 1.71 (SE = 0.52, *p* < 0.05) in the six-month follow-up period, as compared to the six-month pre-enrollment period (i.e., the average marginal effect of the time period indicator in an adjusted model). In the 12 months before and after M3 Team enrollment, participants’ predicted probability of ED use did not change significantly, but the number of predicted ED visits decreased by 2.33 visits (SE = 1.05, *p* < 0.05).

**Table 4:** Adjusted changes in participant emergency department utilization pre- and post-M3 team enrollment

Outcome	Estimate^†^ (SE)
6 months, pre-post change^‡^	
Probability of ED visit	-0.187* (0.086)
Number of ED visits	-1.713* (0.520)
12 months, pre-post change^§^	
Probability of ED visit	-0.093 (0.078)
Number of ED visits	-2.332* (1.051)

*ED* emergency department

*p<0.05, SE=standard error

^†^Estimated average marginal effect retrieved from multilevel mixed-effects generalized linear model with logit link and binomial family for binary outcomes and log link and negative binomial family for count outcomes

^‡^*N*=48, NT=96

^§^*N*=43, NT=86

#### Behavioral health symptom severity

Table 5 presents results from adjusted analyses estimating changes in behavioral health symptom severity scores over time. Controlling for demographic characteristics and other covariates, M3 Team participants experienced consistent decreases in mean BASIS-24 Depression and Functioning subscale scores at 6, 12, and 18 months post-enrollment, compared to baseline scores (*p* < 0.05). At 18 months post-enrollment, this decrease of 0.26 points (SE = 0.10, *p* < 0.05) represented a change from moderate to low symptom severity compared to the unadjusted baseline mean of 1.08 (SD = 0.37). Participants also experienced decreases of nearly 0.06 points (*p* < 0.05) in mean BASIS-24 Self-Harm subscale scores at six and 18 months post-enrollment and a decrease of 0.078 (SE = 0.04, *p* < 0.05) in mean BASIS-24 Emotional Lability subscale core at 12 months post-enrollment. No significant changes in participants’ BASIS-24 Interpersonal Relationships and Psychosis subscale scores were observed during the study period.

**Table 5:** Adjusted changes in participant 24-Item behavior and symptom identification scale and addiction severity index scores following M3 team enrollment

	6-month change from baseline	12-month change from baseline	18-month change from baseline	% change from baseline mean, 18 months
Outcome	Estimate^†^ (SE)	Estimate^†^ (SE)	Estimate^†^ (SE)	
BASIS-24^‡^				
Depression & Functioning	-0.20* (0.08)	-0.237* (0.09)	-0.261* (0.10)	-24%
Interpersonal Relationships	-0.02 (0.02)	-0.016 (0.03)	-0.052 (0.03)	-19%
Self-Harm	-0.06* (0.02)	-0.035 (0.02)	-0.057* (0.03)	-62%
Emotional Lability	-0.05 (0.03)	-0.078* (0.04)	-0.072 (0.04)	-18%
Psychosis	-0.02 (0.02)	-0.011 (0.02)	-0.001 (0.02)	-16%
ASI^§^				
Alcohol Use	-0.10* (0.04)	-0.097* (0.05)	-0.105* (0.05)	-64%
Drug Use	-0.06* (0.02)	-0.065* (0.02)	-0.069* (0.02)	-63%

*SE* standard error, *BASIS-24* 24-Item Behavior and Symptom Identification Scale, *ASI* Addiction Severity Index

*p < 0.05

^†^Estimated average marginal effect retrieved from repeated measures mixed-effects model

^‡^*N*=54, NT=176

^§^*N*=54, NT=174

Participants saw reductions in the mean severity of their substance use symptoms as measured by the ASI (Table 5). Adjusted decreases of approximately 0.1 points in ASI Alcohol Use subscale score were observed at all follow-up time points (*p* < 0.05), a 61% reduction and a change from moderate to low severity relative to the unadjusted baseline mean of 0.16 (SD = 0.28). Similarly, participants experienced decreases of 0.06 (SE = 0.02, *p* < 0.05), 0.07 (SE = 0.02, *p* < 0.05), and 0.07 (SE = 0.02, *p* < 0.05) in mean severity of drug use symptoms as measured by the ASI at six, 12, and 18 months, respectively. These decreases each represent a change from moderate to low severity relative to the baseline mean of 0.11 (SD = 0.13).

## Discussion

Individuals experiencing homelessness with trimorbidity– chronic medical conditions, serious mental illness, and substance use disorders– need tailored care delivery models that comprehensively address their needs. We conducted a retrospective, pre/post analysis of ED utilization and behavioral health outcomes of an initial cohort of 54 individuals enrolled on the M3 Team, an innovative care delivery model providing holistic, integrated, patient- centered care for adults experiencing chronic homelessness with trimorbidity. The inclusion and integration of behavioral health care, physical health care, and support addressing health-related social needs distinguishes the M3 Team from other interventions tailored toward individuals experiencing homelessness who have complex health needs, but that have tended to focus on either behavioral health or physical health care, separately [21].

Results are consistent with other studies of integrated medical care for people experiencing homelessness that have been tested in California, the U.K., Canada, and the U.S. Department of Veterans Affairs [30–33]. M3 Team participants had significant reductions in number of ED visits six and 12 months after enrollment compared to the same time period beforehand and were less likely to have any ED visits in the six months after enrollment compared to the six months prior to enrollment. The magnitude of the adjusted changes in ED use (relative to baseline ED use in the sample) that we observed was large, equivalent to a reduction of nearly 50% from 12 months pre- to post-enrollment. In terms of behavioral health outcomes, M3 Team participants experienced significant reductions in scores over 18 months on both the BASIS-24 and ASI, measuring mental health symptoms and functioning, and alcohol and drug use severity, respectively. Specifically, our adjusted analyses showed reductions in depression and functioning, emotional lability, self-harm, alcohol use symptom severity, and drug use symptom severity equivalent to a shift from moderate to low severity, relative to baseline symptom severity in the sample.

Our analysis does have some limitations. First, our sample size is small, meaning that we may lack sufficient statistical power to detect small changes in outcomes and the generalizability of the care delivery model and outcome findings may be limited. Nonetheless, our study was adequately powered to detect large effect sizes, and we observed a number of significant improvements in both behavioral health symptom severity and ED utilization in this initial analysis of the first cohort of M3 Team participants. We are also planning larger-scale analyses as the number of model participants increases. Second, this is a retrospective, pre/post analysis that lacks a comparison group, and we acknowledge that this threatens the internal validity of the findings. We used a statistical approach that accounts for potential bias due to changes in observable characteristics of the study sample over time. The M3 Team was implemented in response to a dire need in our community and health care ecosystem for this population, and is framed as a pragmatic, implementation-focused demonstration project for this model of care. We hope these preliminary findings will support larger-scale implementation efforts with quasi-experimental or randomized controlled trial designs that will allow for more robust outcome evaluations of this innovative care delivery model.

## Conclusion

The M3 Team is an innovative, integrated, patient-centered model of care for individuals experiencing chronic homelessness with trimorbidity. Compared to baseline, M3 Team participants had reduced ED visits and improved mental health and substance use outcomes. Larger scale interventions are needed to more robustly evaluate outcomes, but this model shows promise for improving the health and well-being of this highly complex and vulnerable population.

## Data Availability

A de-identified dataset can be requested from the last author.

## Abbreviations

M3: Mobile, Medical, and Mental Health Care
ED: Emergency department
BASIS-24: Behavior and Symptom Identification Scale
ASI: Addiction Severity Index
FQHC: Federally qualified health center
HCH: Healthcare for the Homeless
IDDT: Integrated dual disorder treatment
DAST-10: Drug Abuse Screening Test
